# A meta‐analysis of functional group responses to forest recovery outside of the tropics

**DOI:** 10.1111/cobi.12548

**Published:** 2015-06-03

**Authors:** Rebecca Spake, Thomas H. G. Ezard, Philip A. Martin, Adrian C. Newton, C. Patrick Doncaster

**Affiliations:** ^1^ Centre for Biological Sciences, Institute for Life SciencesUniversity of SouthamptonSouthamptonSO17 1BJUnited Kingdom; ^2^ Centre for Conservation Ecology and Environmental Science, Faculty of Science and TechnologyBournemouth UniversityTalbot Campus, Fern BarrowPooleDorsetBH12 5BBUnited Kingdom

**Keywords:** biodiversity offsetting, old‐growth, restoration, secondary forest, bosque primario, bosque secundario, compensación de la biodiversidad, restauración

## Abstract

Both active and passive forest restoration schemes are used in degraded landscapes across the world to enhance biodiversity and ecosystem service provision. Restoration is increasingly also being implemented in biodiversity offset schemes as compensation for loss of natural habitat to anthropogenic development. This has raised concerns about the value of replacing old‐growth forest with plantations, motivating research on biodiversity recovery as forest stands age. Functional diversity is now advocated as a key metric for restoration success, yet it has received little analytical attention to date. We conducted a meta‐analysis of 90 studies that measured differences in species richness for functional groups of fungi, lichens, and beetles between old‐growth control and planted or secondary treatment forests in temperate, boreal, and Mediterranean regions. We identified functional‐group–specific relationships in the response of species richness to stand age after forest disturbance. Ectomycorrhizal fungi averaged 90 years for recovery to old‐growth values (between 45 years and unrecoverable at 95% prediction limits), and epiphytic lichens took 180 years to reach 90% of old‐growth values (between 140 years and never for recovery to old‐growth values at 95% prediction limits). Non‐saproxylic beetle richness, in contrast, decreased as stand age of broadleaved forests increased. The slow recovery by some functional groups essential to ecosystem functioning makes old‐growth forest an effectively irreplaceable biodiversity resource that should be exempt from biodiversity offsetting initiatives.

## Abstract (es)

Un Meta‐Análisis de las Respuestas de Grupos Funcionales a la Recuperación de los Bosques fuera de los Trópicos

**Resumen**

Los esquemas de restauración pasiva y activa de los bosques son utilizados en paisajes degradados en todo el mundo para mejorar el suministro de servicios ambientales y la biodiversidad. La restauración también se implementa cada vez más en los esquemas de compensación de la biodiversidad como retribución de la pérdida del hábitat natural por causa del desarrollo antropogénico. Esto ha causado preocupaciones sobre el valor del reemplazo de los bosques primarios por plantaciones, lo que motiva a la investigación sobre la recuperación de la biodiversidad durante la edad de pie del bosque. La diversidad funcional ahora es propuesta como una clave métrica para el éxito de la restauración, pero ha recibido muy poca atención analítica a la fecha. Realizamos un meta‐análisis de 90 estudios que midieron las diferencias en la riqueza de especies para grupos funcionales de hongos, líquenes y escarabajos entre bosques primarios de control y bosques sembrados o de tratamiento secundario en las regiones templadas, boreales y mediterráneas. Identificamos las relaciones específicas de grupos funcionales en la respuesta de la riqueza de especies a la edad de pie después de la perturbación del bosque. Los hongos ectomicorrízicos necesitaron en promedio de 90 años para recuperar sus valores de bosque promedio (entre 45 años e irrecuperables al 95% de los límites de predicción) y los líquenes epífitos necesitaron de 180 años para alcanzar el 90% del valor de bosque primario (entre 140 años y nunca para la recuperación de valores de bosque primario al 95% de los límites de predicción). La riqueza de escarabajos no saproxílicos, al contrario, disminuyó conforme incrementó la edad de pie de los bosques de hojas anchas. La lenta recuperación por parte de algunos grupos funcionales esenciales para los servicios ambientales hace del bosque primario un recurso irremplazable de biodiversidad que debería estar exento de las iniciativas de compensación de la biodiversidad.

## Introduction

The world's forests contain over 80% of global terrestrial biodiversity (Aerts & Honnay 2011) and contribute crucial ecosystem services including carbon storage and protection of watersheds, fisheries, and soils (MA 2005). Rates of deforestation are alarmingly high (FAO 2010); 5.2 million ha of global forest area were lost each year from 2000 to 2010 (FAO 2010). Currently, just 12% of global forest cover has biodiversity conservation designated as its primary function (FAO 2010). Although these protected reserves are essential to national and international strategies to counter biodiversity loss, they are insufficient to conserve forest biodiversity because they are too few, too isolated, and too inadequately protected from over‐exploitation (Fischer et al. 2006; Lindenmayer et al. 2006). Therefore, conservation efforts are increasingly implementing forest restoration measures worldwide, both within and outside reserves, to enhance biodiversity and ecosystem service provision in degraded landscapes (Chazdon 2008; Benayas et al. 2009; Bullock et al. 2011). At the 11th Convention of the Parties, the Convention on Biological Diversity declared that ecological restoration and rehabilitation are crucial for the recovery of biological diversity and critical ecosystem services.

Forest restoration measures range from passive restoration, involving natural or unassisted forest recovery following the removal of environmental stressors such as grazing, to active restoration, involving human interventions such as planting to accelerate and influence the successional trajectory of recovery (Benayas et al. 2009; Holl & Aide 2011; Morrison & Lindell 2011). Forest restoration is also used as a biodiversity offsetting mechanism for mitigating the loss of natural area incurred by development. There are two major types of biodiversity offsets: restoration offsets and protection offsets. Restoration offsets aim to generate new habitat in an offset site to compensate for the loss of habitat due to development at the impact site. Protection offsets involve protecting existing biodiversity from further threats such as deforestation (Maron et al. 2012; Curran et al. 2014). The primary goal of biodiversity offsetting is to prevent change to species composition and habitat structure and to preserve ecosystem function or perceived cultural value associated with biodiversity (Bull et al. 2013).

A major criticism of restoration offset practice concerns the existence of time lags between the implementation of restoration action and the accrual of the intended benefits (Bull et al. 2013). Several meta‐analyses have quantified the recovery times required for biodiversity, including measures of species diversity and composition, to reach equivalence to some reference state. The reference state typically has attributes of an undegraded ecosystem (Bullock et al. 2011) characterized by relatively undisturbed old‐growth forest. The majority of meta‐analyses of stand age and biodiversity relationships have been produced for taxonomic groups including trees, epiphytes, birds, amphibians, mammals, ants, and other invertebrates in secondary tropical forests (Dunn 2004; Chazdon et al. 2009; Martin et al. 2013). Fewer syntheses exist for forest recovery outside the tropics. A recent global synthesis by Curran et al. (2014) predicted century‐long recovery times in species richness and composition within broad taxonomic groups including plants, trees, mammals, birds, herpetofauna, and invertebrates for naturally regenerating secondary forests in temperate, boreal, and tropical biomes. These syntheses indicate that different taxonomic groups exhibit contrasting patterns and rates of recovery over time (e.g., Dunn 2004; Chazdon et al. 2009; Curran et al. 2014). This must be recognized in forest management strategies because different taxa make different contributions to ecosystem functioning (Hooper et al. 2002; Dirzo et al. 2014).

Syntheses to date have focused on charismatic taxa in tropical biomes. Evaluation of restoration objectives often pivots on the recovery of assemblages across broad taxonomic groups. The success of restoration programs, however, is being evaluated increasingly through assessments of functional diversity and critical ecosystem functions (Aerts & Honnay 2011; Audino et al. 2014). In particular, one of the nine core success criteria suggested by the Society for Ecological Restoration is the representation of “all functional groups necessary for the continued development and/or stability of the restored ecosystem” (SER 2004). Distinguishing among the responses of different functional groups within broad groupings of taxa can facilitate an understanding of the mechanisms that underlie community responses to environmental change and determine ecosystem functioning (Diaz et al. 2007; Lavorel et al. 2008; Laliberte et al. 2010).

We assessed the recovery of functional groups in restored forests outside the tropics, in temperate, boreal, and Mediterranean regions. We focused on lichens, fungi, and beetles because of their underrepresentation in existing quantitative syntheses of forest biodiversity recovery (Dunn 2004; Chazdon et al. 2009; Curran et al. 2014). These taxa are well studied, relatively species rich, and sensitive to stand‐level processes, and their communities perform vital functions in forest ecosystems. Lichens contribute to forest water and nutrient cycles through precipitation interception and nutrient sequestration (Knops et al. 1996; Ellis 2012); fungi are the main agents of wood decomposition and thus carbon and nutrient cycling, and they form mycorrhizal associations with trees (Crockatt 2012); beetle functional roles include herbivory, predation, decomposition, and microhabitat creation (Buse & Good 1993; Barton et al. 2009). We differentiated functional groups by resource acquisition to reflect dependencies on resources or conditions that peak at different stages during forest recovery. For example, deadwood‐dependent taxa were expected to benefit from forest succession because deadwood generally increases in volume and diversity as a stand ages (Humphrey et al. 2003). Furthermore, classification by resource acquisition, a process central to most biotic interactions, captures variation that is relevant to relationships between biodiversity and ecosystem functioning (Flynn et al. 2009).

## Methods

### Systematic Review Scope

We followed standard systematic review methods (Pullin & Stewart 2006) to collate empirical studies from temperate and boreal forests that compared biodiversity in planted or secondary forest with old‐growth, primary, or mature controls. Temperate, boreal, and Mediterranean forest was defined as forest lying outside the –40° to +40° latitudinal band. Secondary forests (our treatment forests) had to have originated by planting or natural regeneration following major, stand‐replacing disturbance including clearcutting and catastrophic wildfire. Controls had to have had little to no management over the past 50 years. Because passive restoration involves natural succession, studies that measured biodiversity at different stages of natural succession following disturbance were relevant to this analysis. Relevant studies published between 1970 and March 2015 were identified through literature searches in the ISI Web of Science. We used search terms relating to the focal taxa, forest type, and species richness data (see Supporting Information for the search query). Species richness was used as a proxy for biodiversity because species richness is the simplest and most widely used biodiversity measure (Magurran 2004). For those studies that reported data in figures only, numerical information was extracted using DataThief (Tummers 2006).

To ensure biologically meaningful comparisons, publications had to satisfy strict inclusion criteria. Treatment and control forest stands had to have similar composition of canopy dominants. Almost all of the collated studies featured treatment‐control comparisons within observational chronosequences. Studies reporting only before–after comparisons were excluded because they lacked a true control (Duguid & Ashton 2013). In agreement with Hurlbert's (1984) classification of acceptable and unacceptable study designs, we included studies that were definitively free of simple pseudoreplication so as to avoid spurious differences from confounding treatment variation with random site variation. We included studies that had replicate treatment forests spatially interspersed with replicates of control forests. Studies therefore had either completely randomized, randomized block, or systematic study designs (Hurlbert 1984).

Beetles, lichens, and fungi were assigned to functional groups according to resource acquisition. Beetles were categorized as saproxylic (species that depend on deadwood during some part of their life cycle [Speight 1989] or non‐saproxylic (groups not explicitly defined as saproxylic, e.g., ground beetles). Fungi were characterized as saprotrophic on deadwood, saprotrophic on litter, parasitic, or ectomycorrhizal (Ferris et al. 2000; Humphrey et al. 2003). Lichens were categorized as epiphytic (species that grow on the bark of trees) or terricolous (species growing on soil).

### Statistical Analyses

For each biodiversity comparison, the log response ratio (ln*R*) of species richness was calculated between secondary forest (treatment group) and old‐growth forest (control group):

(1) In R= In (x¯2)− In (x¯1),

where $\left({\bar{x}}_{2}\right)$ is the mean species richness of treatment forest stands and $\left({\bar{x}}_{1}\right)$ is the mean species richness of old‐growth stands. The ln*R* describes the proportional difference in species richness between control and treatment groups. The natural log transformation of the response ratio both linearizes the metric, treating deviations in the denominator and the numerator as equal, and normalizes its otherwise skewed distribution (Hedges et al. 1999).

All statistical analyses and calculations were performed in R (version3.1.1) (R Core Team 2014). Publication bias may be suspected if small positive effect sizes are present without small negative effect sizes (Newton et al. 2009). We tested this in the METAFOR package (Viechtbauer 2010) by assessing a funnel plot of effect size versus standard error of the effect size (Sterne & Egger 2001) (output in Supporting Information). Weighted regression with multiplicative dispersion and standard error as the predictor did not detect funnel plot asymmetry, (*t*_88_ = –0.53, *p* = 0.54), indicating no evidence of publication bias.

To quantify how the species richness of different functional groups varies with stand age, we constructed linear mixed models containing an interaction between stand age and functional group. Latitude and transition categories, which are consistently reported in the literature, were added to the model. Transitions included clearcut to planted, clearcut to regenerated, fire to planted, and fire to regenerated. Treatment forest stands were either managed or unmanaged, where managed forests were secondary forests from which many trees had been removed (e.g., thinning since initial planting or regeneration). We included quadratic or log_10_ relationships and stand age to test for possible nonlinear biodiversity recovery with stand age. To account for possible pseudoreplication from multiple biodiversity comparisons (studies) per observational chronosequence, each model included chronosequence as a random factor.

Meta‐analyses may weight study‐wise effect sizes to improve precision of the estimate of overall mean effect and the power of tests (Gurevitch & Hedges 1999). Effect sizes are commonly weighted by the inverse of within‐group variance to raise the relative contributions of studies with lower unmeasured variation, on the principle that these will have higher precision (Koricheva & Gurevitch 2014). In the absence of a suitable measure of within‐group variation being provided by primary studies, some meta‐analyses weight by sample size, on the principle that variance is expected to decrease with sample size, all else being equal. We did not weight effect sizes because of two issues relating to variance estimation and sample size that occur frequently in ecological study design (see Supporting Information for reasoning). Differences between weighted and unweighted statistics are generally small for meta‐analysis (Cardinale et al. 2006; Marvier et al. 2007; Benayas et al. 2009). Furthermore, unweighted meta‐regression is often more robust because it does not use potentially misleading estimation of error variances (Fletcher & Dixon 2012).

All possible additive models were constructed using maximum likelihood methods in package MuMIn (Barton 2013). Power was insufficient to test for interactions other than stand age*functional group. We used Akaike's information criterion (AIC) with small‐sample correction (AICc) to identify support for each model (Burnham & Anderson 2002). The AICc gives a parsimonious quantification of model fit by incorporating both deviance explained and number of parameters used. Fit of selected models was assessed by calculating marginal *R* ^2^ following Nakagawa and Schielzeth (2013). Latitudes were centered to improve the interpretability of regression coefficients (Schielzeth 2010). Graphics were produced using ggplot2 (Wickham 2009), with ln*R* values transformed to show change more intuitively as percentage difference from old‐growth forest stands. Planned orthogonal contrasts were applied to the best model (with the lowest AICc value) in order to interpret differences among functional groups and their interaction with stand age (Doncaster & Davey 2007).

## Results

The literature search yielded 3810 publications. Of these, 47 satisfied inclusion criteria concerning study taxa and latitude and appropriateness of control and treatment stands. Fifteen of these had unclear or pseudoreplicated study designs. We included the remaining 33 publications in the analysis (Supporting Information). These provided 90 separate biodiversity comparisons, hereafter referred to as studies (Table 1). Of these studies, 40 (44%) were from Europe, 45 (50%) from North America, and 5 (5%) from Asia and Australia (Table 1 & Supporting Information). Biases existed in terms of the forest type and the functional groups investigated. Of the 90 studies, 19 (21%) were from broadleaved forest, and 13 of these 19 were on non‐saproxylic beetles. The non‐saproxylic beetle group was therefore divided into broadleaved and coniferous subgroups. Authors indicated some degree of harvesting (e.g., thinning operations) in the treatment stands of 5 out of 90 studies. The influence of management was therefore not assessed. The single study on terricolous lichens that satisfied inclusion criteria was grouped with epiphytic lichens. No suitable data were found on parasitic fungi.

**Table 1:** Geographic origin and focal functional groups of studies used in the meta‐analysis of functional group richness recovery with stand age

	Number of studies by continent				
Group	N. America	Europe	Asia	Australia	Total
Epiphytic lichens	12	7	0	0	19
Ectomycorrhizal fungi	14	3	0	0	17
Deadwood fungi	5	7	0	0	12
Litter fungi	3	4	0	0	7
Saproxylic beetles	3	5	2	0	10
Non‐saproxylic beetles	8	14	1	2	25
Total	45	40	3	2	90

The minimum adequate model selected to explain recovery of species richness in secondary forests included functional group (*F*_6,45_ = 8.92, *p* < 0.001) and log_10_ stand age (*F*_1,45_ = 5.91, *p* = 0.019), their interaction (*F*_6,45_ = 4.41, *p* < 0.002; Table 2), and latitude (*F*_1,30_ = 1.75, *p* = 0.196; Table 2). This model had the lowest AICc score (the next best model had ∆AICc 4.9) and explained 56% of the variation among studies (Table 2). Transition category did not feature in the best model. Planned orthogonal contrasts revealed significant differences in recovery between broadleaved non‐saproxylic beetles and pooled coniferous saproxylic and non‐saproxylic beetles and between saproxylic and coniferous non‐saproxylic beetle groups (Table 3).

**Table 2:** Variables included in linear mixed models developed to explain variation in the log response ratio of species richness in planted and secondary forest stands relative to old‐growth forest

	Variables in model^b^								
Model^a^	group	log_10_(age)	group*log_10_(age)	lat	trans	df	AICc	∆AICc	Marginal *R* ^2^
Null						3	94.81	20.01	0.17
1	+	+	+	+		17	74.80	0.00	0.56
2	+	+	+			16	79.70	4.90	0.54
3	+	+	+	+	+	20	80.83	6.03	0.58

a In addition to the null model, only models with ∆AICc < 7 are shown (i.e., those with considerable support [Burnham & Anderson 2002]).

b Abbreviations: group, functional groups comprising lichens, ectomycorrhizal fungi, litter fungi, deadwood fungi, saproxylic beetles, and non‐saproxylic beetles in coniferous and broadleaved forest; log_10_(age), log_10_ of stand age in years; lat, centered latitude; trans, transition category representing the origin of the treatment stands, including clearcut planted, clearcut secondary, fire planted, and fire secondary.

**Table 3:** Planned orthogonal contrasts among 7 functional groups in the best model of species‐richness recovery in planted and secondary forests.*

	Main effect of group		Interaction with log_10_(age)	
Comparison	*t*	*p*	*t*	*p*
Coniferous and broadleaved non‐saproxylic beetles and saproxylic beetles vs. all other groups	–0.59	0.555	–0.06	0.952
Broadleaved non‐saproxylic beetles vs. pooled coniferous non‐saproxylic beetles and saproxylic beetles	5.65	<0.001	–3.93	<0.001
Saproxylic beetles vs. coniferous non‐saproxylic beetles	–3.59	<0.001	2.45	0.018
Lichens vs. pooled coniferous litter, deadwood and ectomycorrhizal fungi	0.87	0.390	–0.74	0.464
Litter fungi vs. pooled deadwood and ectomycorrhizal fungi	1.26	0.215	–0.77	0.445
Deadwood fungi vs. ectomycorrhizal fungi	–1.40	0.168	1.20	0.237

*All comparisons had df = 45. Negative *t* values indicate lower coefficients for the first group than the second group, and positive values indicate higher coefficients for the first group than the second group.

**Figure 1:**
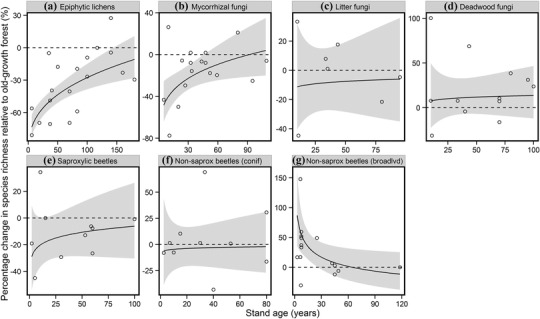
Influence of stand age on percent change in species richness for 7 functional groups in planted and secondary forest relative to old‐growth forest stands (horizontal dashed line, no difference between undisturbed old‐growth forest and treatment [planted and secondary] forest stands; gray, 95% prediction intervals based on uncertainty in fixed effects only; saprox, saproxylic; conif, coniferous; broadlvd, broadleaved). Regressions had coefficients of the best model based on AIC_c_. Latitude was fixed at its mean value for all predictions.

Different functional groups showed different directions and rates of recovery following disturbance. For ectomycorrhizal fungi, a best estimate of recovery to undisturbed old‐growth values of species richness was 90 years (between 45 years and unrecoverable at 95% prediction limits) (Fig. 1). The best estimate for lichens was 180 years to reach 90% of undisturbed forest values (between 140 years and never for full recovery) (Fig. 1). Saproxylic beetles had a best estimate of about 60 years to reach 90% of old‐growth values (between 10 years and never for full recovery). In coniferous forest, non‐saproxylic beetle species richness did not differ detectably between control and treatment forest. In broadleaved forest by contrast, non‐saproxylic beetle species richness appeared to benefit from early successional stages; treatment forest exhibited about twice (from 1.4 to 2.5 times) the species richness of old‐growth forest immediately following major disturbance. Deadwood and litter fungi species richness did not differ detectably between old‐growth and treatment stands (Fig. 1).

## Discussion

### Recovery of Species Richness of Functional Groups

We found functional‐group–specific relationships between species richness and stand age following forest disturbance. Lichen, ectomycorrhizal fungi, and saproxylic beetle richness was much lower in early successional or young planted forest than undisturbed old‐growth forest (Fig. 1). Recovery to old‐growth values of species richness required 90 years for ectomycorrhizal fungi, 60 years for saproxylic beetles, and >100 years for lichens. Non‐saproxylic broadleaved beetle communities benefited from major disturbance; early successional forest contained around twice the species richness of undisturbed forest (1.4–2.5 times; Fig. 1). Our result of functional group‐specific responses to stand age extended this pattern from tropical multi‐taxon syntheses. Dunn (2004) synthesized data across a wide range of animal taxa, including bats, birds, and invertebrates, and found that faunal species richness in tropical secondary forest can rapidly resemble that of old‐growth forest within just 20–40 years following major disturbance. Martin et al. (2013) found that tropical epiphytic plants took considerably longer; over 100 years were needed for species richness to recover in forest regenerating from agricultural clearance.

The increase in lichen richness with stand age is likely attributable to the combined effects of time, which favored colonization by dispersal‐limited species, and changes in substrate conditions associated with tree ageing (increased surface availability, changes in bark pH and texture, and increased stable substrate due to reduced growth rates) (Nascimbene et al. 2013). Johansson (2008) investigated lichen–stand age relationships in boreal chronosequences by meta‐regressing stand age with the species richness of forest age class as a proportion of the total species pool richness (all age classes combined). Using this approach, no relationship was found between proportional richness and stand age, which is likely due to compositional differences between younger and older stands (Johansson 2008). Ectomycorrhizal fungi form mutualistic symbioses with tree hosts by forming a sheath around the root tip of the tree that exchanges soil‐derived nutrients for carbohydrates from host trees (Smith & Read 2008). Ectomycorrhizal diversity is expected to increase with stand age (Ferris et al. 2000; Humphrey et al. 2000), as found in this study (Fig. 1), in response to increasing density of tree roots, leaf area (Simard & Durall 2004), and associated carbon availability for ectomycorrhizal partners (Twieg et al. 2007). Deadwood‐dependent richness, including deadwood fungi and saproxylic beetles, is expected to rise with stand age, owing to the increase in deadwood volume and decay stage over time, and therefore as a function of the species–area relationship and provision of diverse microhabitats (Heilmann‐Clausen & Christensen 2004; Lonsdale et al. 2008). Although saproxylic beetle richness increased steadily as stand age increased, species richness of deadwood fungi differed little between old‐growth controls and planted and secondary forest (Fig. 1). Little deadwood is produced in young forest stands, and its presence in these studies may have been a product of the major disturbance event that initiated the stand or due to deadwood created through self‐thinning of young stands. Higher deadwood volumes in young treatment stands may therefore be responsible for the comparable richness of deadwood fungi in treatment and old‐growth stands.

Studies investigating the succession of non‐saproxylic beetle groups attribute higher biodiversity values in early successional forest to high numbers of open‐habitat and generalist species, favored by conditions afforded by open canopies (da Silva et al. 2008; Taboada et al. 2008). Although we did not examine compositional differences between treatment and old‐growth forest, this may be the case here for broadleaved non‐saproxylic beetles (Fig. 1), which decreased in richness as stand age increased (see also Lange et al. 2014). Coniferous non‐saproxylic beetle richness differed little between old‐growth and treatment stands. More data are needed to understand compositional differences that might explain this pattern.

The inclusion of latitude improved the goodness of fit and explanatory power of the best model (Table 2). Latitude is a coarse proxy for changes among many local environmental descriptors. For example, high‐latitude soils generally contain fewer nutrients than low‐latitude soils (Zvereva et al. 2008), and latitude can alter the slope of species–area relationships on islands (Solymos & Lele 2012). Furthermore, spatial continuity of forest at the landscape scale is less likely to be a limiting factor for dispersal in widely forested regions such as boreal Fennoscandia, as opposed to other areas in temperate western Europe where forests have been reduced to smaller remnants (Parviainen et al. 1999). Further identification of latitudinal components that cause species richness differences between old‐growth and treatment forest is problematic because of the high co‐linearity among the components that underpin the broad latitudinal gradient.

We found that functional groups within broad taxonomic groups exhibited varying responses to forest recovery (Fig. 1). This might suggest that previous meta‐analyses investigating biodiversity variation relative to environmental variables in which organisms were classified into uniform taxonomic groups may have undervalued some patterns of biodiversity‐by‐environment relationships. We found that functional groups were not equivalent to broad taxonomic groups for fungi and beetles (Fig. 1). For example, pooling saproxylic and non‐saproxylic beetles, which showed contrasting responses to stand age, may wrongly suggest that forest stand age has negligible effects on beetle biodiversity, which was clearly not the case.

### Knowledge Gaps

Our systematic review yielded just 33 publications (90 individual studies) in which old‐growth was compared with planted or secondary forests in a statistically robust way. For some functional groups, this led to small sample sizes and low precision in ln*R* values (Fig. 1). The small number of publications suggests a continuing lacuna of empirical data for evaluating biodiversity indicators. Sustainable forest management requires effective biodiversity indicators for monitoring (Lindenmayer et al. 2000), and there is therefore an urgent need for more carefully designed studies to identify and evaluate such indicators. Of the 90 suitable studies, 79% were conducted in coniferous forest. More data are needed from broadleaved successional chronosequences, which are underrepresented in the literature.

### Conservation Implications

The primary goal of biodiversity offsetting is to achieve no net loss of biodiversity. Our results show that through restoration offsetting, this goal is unachievable within a reasonable time frame. Functional groups in secondary forest require over a century for lichens and almost a century for ectomycorrhizal fungi to recover species richness values equivalent to old‐growth forest (Fig. 1). The slow recovery of species richness for some functional groups essential to ecosystem functioning makes old‐growth forest an effectively irreplaceable biodiversity resource that should be exempted from restoration offset initiatives. Interim losses of old‐growth forest from landscapes over century‐long time scales disable their function as biodiversity donors to developing forests, lead to the loss of functional groups, and jeopardize ecosystem function (Wardle & Zackrisson 2005).

Our results support the findings of Curran et al. (2014), who also demonstrated long recovery times in their global analysis across broad taxonomic groupings in secondary forests. They found that species richness converges to old‐growth reference values within a century, species similarity takes about twice as long, whilst assemblage composition takes up to an order of magnitude longer (hundreds to thousands of years). Our finer‐scale analysis showed significant differences in the responses of different functional groups within broad taxonomic groupings and century‐long recovery times for some functional groups.

These results support the value of protecting old‐growth forest through reserve creation, set‐aside of overmature stands for biodiversity conservation, and implementation of schemes that extend rotation‐length of secondary forests within production forest landscapes. Examples of the latter include woodland key habitats (WKHs) and green tree retention (GTR) practices in Fennoscandia and the creation of temporary “ageing islands” in French high forests (Lassauce et al. 2013).

Our observation of varying responses of different functional groups to forest recovery has important implications not only for restoration initiatives but also for sustainable forest management of productive forests. Sustainable forest management represents a paradigm of forest management strategies that balance timber provision with the production of other goods and services that human society needs through the integration biodiversity conservation within productive forest landscapes (Lindenmayer et al. 2012). Forest successional stages that support different functional groups must be represented in production landscapes because different groups make different contributions to ecosystem functioning (Hooper et al. 2002).

Our results apply to the scale of forest stands and therefore to alpha diversity. With different functional groups exhibiting contrasting species richness levels in different successional stages, it is possible that diversity at the landscape scale may be higher in a mosaic of lower richness patches than in a homogeneous landscape with higher alpha diversity (Duguid & Ashton 2013). For example, the decline of broadleaved non‐saproxylic beetles as stand age increased (Fig. 1) supports suggestions that young secondary forest must be included in managed forest mosaics for invertebrate conservation (de Warnaffe & Lebrun 2004).

## Supporting information

Disclaimer: Supplementary materials have been peer‐reviewed but not copyedited.

The keyword search (Appendix S1), list of publications included in the meta‐analysis and the geographic distribution of their study (Appendix S2 and S3, respectively), a funnel plot (Appendix S4), and the justification for presenting an unweighted regression (Appendix S5) are available online. The authors are solely responsible for the content and functionality of these materials. Queries (other than absence of the material) should be directed to the corresponding author.
