# Supplementary material for: A meta‐analysis of functional group responses to forest recovery outside of the tropics
Source: Conserv Biol. 2015 Jun 3;29(6):1695–703. doi: 10.1111/cobi.12548 (PMC4973697; doi:10.1111/cobi.12548)
Supplement: Supplementary file 1 — The keyword search (Appendix S1), list of publications included in the meta‐analysis and the geographic distribution of their study (Appendix S2 and S3, respectively), a funnel plot (Appendix S4), and the justification for presenting an unweighted regression (Appendix S5) are available online. The authors are solely responsible for the content and functionality of these materials. Queries (other than absence of the material) should be directed to the corresponding author. [file COBI-29-1695-s001.docx]

**Supporting Information**

Details of methods, statistics and studies used in the analysis.

### Appendix S1 - Keywords used for search in ISI Web of Knowledge

Relevant studies were identified through computerised searches in the ISI Web of Science database. The finalised search query performed on 10 March 2015 was:

TS = (fung* OR macrofung* OR myco* OR wood-decay OR sapro* OR parasit* OR pathogenic* OR ectomycorrhiz* OR arbusc* OR endomycorrhiz* OR mycorrhiz* OR basidiomyc* OR ascomyc OR lichen* OR cyanobacteria* OR epiphyt* OR coleoptera* OR beetle OR carabid* OR saproxylic OR ground-beetle)

AND

TS = (forest* OR woodland OR wooded)

AND

TS = (richness OR divers* OR biodivers*)

AND

TS = (ancient OR old-growth OR primary OR virgin OR pristine OR mature OR remnant OR “ecological continuity” OR “habitat continuity” OR “forest continuity” OR “environmental continuity” OR “historical continuity” OR “stand age” OR “forest age” OR chronosequence OR succession* OR plantation* OR secondary OR clear-cut* OR clearcut* OR clear-fell* OR clearfell* OR afforest* OR “planted forest” OR “forest cycle” OR stand structur* OR multi-age OR “multiple aged” OR “demographic transition” OR “canopy closure” OR “stem exclusion” OR “over mature” OR “stand initiation” OR “understorey reinitiation” OR “pole stage” OR pre-thicket OR mid-rotation OR rotation)

NOT

TS = (mangrove OR bushland)

### Appendix S2 – Studies included in the analysis

Baker, S. C. 2006. A comparison of litter beetle assemblages (Coleoptera) in mature and recently clearfelled Eucalyptus obliqua forest. Australian Journal of Entomology 45:130-136.

Boudreault, C., Y. Bergeron, S. Gauthier, and P. Drapeau. 2002. Bryophyte and lichen communities in mature to old-growth stands in eastern boreal forests of Canada. Canadian Journal of Forest Research-Revue Canadienne De Recherche Forestiere 32:1080-1093.

Cardenas, A. M., and C. Buddle. 2009. Introduced and native ground beetle assemblages (Coleoptera: Carabidae) along a successional gradient in an urban landscape. Journal of Insect Conservation 13:151-163.

Countess, R. E., B. Kendrick, and J. A. Trofymow. 1998. Macrofungal diversity in successional Douglas-fir forests. Northwest Science 72:110-112.

Cunningham, S. A., R. B. Floyd, and T. A. Weir. 2005. Do Eucalyptus plantations host an insect community similar to remnant Eucalyptus forest? Austral Ecology 30:103-117.

de Warnaffe, G. D., and M. Dufrene. 2004. To what extent can management variables explain species assemblages? A study of carabid beetles in forests. Ecography 27:701-714.

Dettki, H., and P. A. Esseen. 1998. Epiphytic macrolichens in managed and natural forest landscapes: a comparison at two spatial scales. Ecography 21:613-624.

Dollin, P. E., C. G. Majka, and P. N. Duinker. 2008. Saproxylic beetle (Coleoptera) communities and forest management practices in coniferous stands in southwestern Nova Scotia, Canada. Zookeys:291-336.

Duchesne, L. C., R. A. Lautenschlager, and F. W. Bell. 1999. Effects of clear-cutting and plant competition control methods on carabid (Coleoptera : Carabidae) assemblages in northwestern Ontario. Environmental Monitoring and Assessment 56:87-96.

Dunham, S. M., K. H. Larsson, and J. W. Spatafora. 2007. Species richness and community composition of mat-forming ectomycorrhizal fungi in old- and second-growth Douglas-fir forests of the HJ Andrews Experimental Forest, Oregon, USA. Mycorrhiza 17:633-645.

Dynesius, M., and U. Zinko. 2006. Species richness correlations among primary producers in boreal forests. Diversity and Distributions 12:703-713.

Enns, K.A, Trofymow, J.A. and Goodman, D.M. 1999. Arboreal lichens in successional forests of southern Vancouver Island. Canadian Forest Service Forest Ecosystem Processes Network. Pacific Forestry Centre, Victoria, B.C. Inf. Rep. BC-X-382. 17p.

Ferris, R., A. J. Peace, and A. C. Newton. 2000. Macrofungal communities of lowland Scots pine (Pinus sylvestris L.) and Norway spruce (Picea abies (L.) Karsten.) plantations in England: relationships with site factors and stand structure. Forest Ecology and Management 131:255-267.

Fuller, R. J., T. H. Oliver, and S. R. Leather. 2008. Forest management effects on carabid beetle communities in coniferous and broadleaved forests: implications for conservation. Insect Conservation and Diversity 1:242-252.

Johansson, T., J. Hjalten, J. Hilszczanski, J. Stenlid, J. P. Ball, O. Alinvi, and K. Danell. 2007. Variable response of different functional groups of saproxylic beetles to substrate manipulation and forest management: Implications for conservation strategies. Forest Ecology and Management 242:496-510.

Jukes, M. R., A. J. Peace, and R. Ferris. 2001. Carabid beetle communities associated with coniferous plantations in Britain: the influence of site, ground vegetation and stand structure. Forest Ecology and Management 148:271-286.

Junninen, K., M. Simila, J. Kouki, and H. Kotiranta. 2006. Assemblages of wood-inhabiting fungi along the gradients of succession and naturalness in boreal pine-dominated forests in Fennoscandia. Ecography 29:75-83.

Kranabetter, J. M., J. Friesen, S. Gamiet, and P. Kroeger. 2005. Ectomycorrhizal mushroom distribution by stand age in western hemlock - lodgepole pine forests of northwestern British Columbia. Canadian Journal of Forest Research-Revue Canadienne De Recherche Forestiere 35:1527-1539.

Lassauce, A., Larrieu, L., Paillet, Y., Lieutier, F. & Bouget, C. 2013. The effects of forest age on saproxylic beetle biodiversity: implications of shortened and extended rotation lengths in a French oak high forest. Insect Conservation and Diversity, 6: 396-410.

Lindner, D. L., H. H. Burdsall, and G. R. Stanosz. 2006. Species diversity of polyporoid and corticioid fungi in northern hardwood forests with differing management histories. Mycologia 98:195-217.

Makino, S., H. Goto, M. Hasegawa, K. Okabe, H. Tanaka, T. Inoue, and I. Okochi. 2007. Degradation of longicorn beetle (Coleoptera, Cerambycidae, Disteniidae) fauna caused by conversion from broad-leaved to man-made conifer stands of Cryptomeria japonica (Taxodiaceae) in central Japan. Ecological Research 22:372-381.

Marmor, L., T. Torra, L. Saag, and T. Randlane. 2011. Effects of forest continuity and tree age on epiphytic lichen biota in coniferous forests in Estonia. Ecological Indicators 11:1270-1276.

McGeoch, M. A., M. Schroeder, B. Ekbom, and S. Larsson. 2007. Saproxylic beetle diversity in a managed boreal forest: importance of stand characteristics and forestry conservation measures. Diversity and Distributions 13:418-429.

Neitlich, P. 1993. Lichen abundance and biodiversity along a chronosequence from young managed stands to ancient forest. Masters Thesis, University of Vermont.

Paquin, P. 2008. Carabid beetle (Coleoptera : Carabidae) diversity in the black spruce succession of eastern Canada. Biological Conservation 141:261-275.

Radies, D. N., and D. S. Coxson. 2004. Macrolichen colonization on 120-140 year old Tsuga heterophylla in wet temperate rainforests of central-interior British Columbia: a comparison of lichen response to even-aged versus old-growth stand structures. Lichenologist 36:235-247.

Rheault, H., L. Belanger, P. Grondin, R. Ouimet, C. Hebert, and C. Dussault. 2009. Stand composition and structure as indicators of epixylic diversity in old-growth boreal forests. Ecoscience 16:183-196.

Sippola, A. L., Siitonen, J. & Punttila, P. 2002. Beetle diversity in timberline forests: a comparison between old-growth and regeneration areas in Finnish Lapland. Annales Zoologici Fennici, 39, 69-86.

Smith, J. E., R. Molina, M. M. P. Huso, D. L. Luoma, D. McKay, M. A. Castellano, T. Lebel, and Y. Valachovic. 2002. Species richness, abundance, and composition of hypogeous and epigeous ectomycorrhizal fungal sporocarps in young, rotation-age, and old-growth stands of Douglas-fir (Pseudotsuga menziesii) in the Cascade Range of Oregon, USA. Canadian Journal of Botany-Revue Canadienne De Botanique 80:186-204.

Stenbacka, F., J. Hjalten, J. Hilszczanski, and M. Dynesius. 2010. Saproxylic and non-saproxylic beetle assemblages in boreal spruce forests of different age and forestry intensity. Ecological Applications 20:2310-2321.

Taboada, A., D. J. Kotze, R. Tarrega, and J. M. Salgadoa. 2008. Carabids of differently aged reforested pinewoods and a natural pine forest in a historically modified landscape. Basic and Applied Ecology 9:161-171.

Timm, A., J. Buse, T. Dayan, W. Hardtle, T. Levanony, and T. Assmann. 2009. At the interface of historical and present-day ecology: ground beetles in woodlands and open habitats in Upper Galilee (Israel) (Coleoptera: Carabidae). Zoology in the Middle East 47:93-104.

Twieg, B. D., D. M. Durall, and S. W. Simard. 2007. Ectomycorrhizal fungal succession in mixed temperate forests. New Phytologist 176:437-447.

### Appendix S3 - Distribution of studies used in the analysis. Point size represents the number of studies per 4 degree grid cell.

###
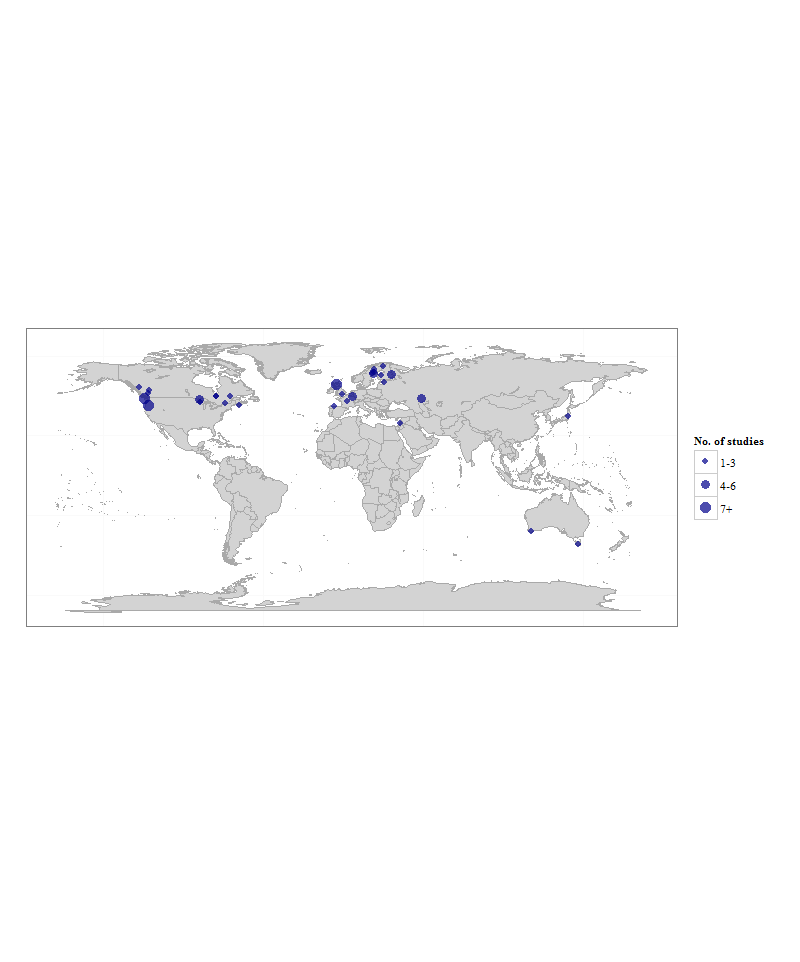


### Appendix S4 - Funnel plot of the log response ratio and standard error of the entire dataset. An absence of publication bias is indicated by a symmetric funnel with larger spread at smaller sample sizes; the variation around the effect size should decrease as sample size increases.

###
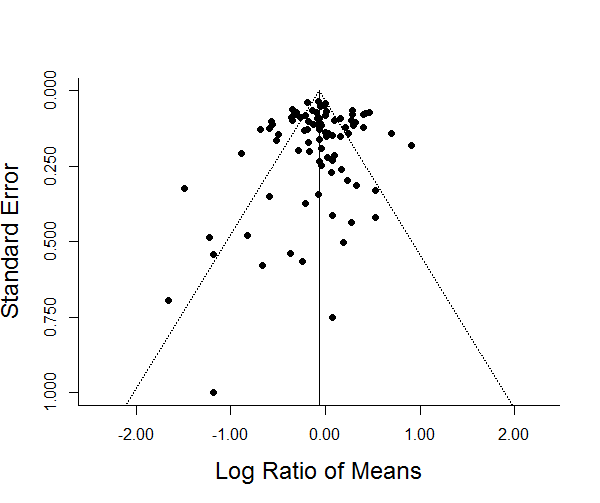


### Appendix S5 – Rationale for unweighted regression

Here we detail issues with weighting our meta-regressions to improve precision, which may arise frequently in ecological experimental design.

In weighting by the inverse of within-group variance, the variance calculation depends on whether studies have completely randomised or matched designs ([Borenstein et al. 2009](#_ENREF_2); [Lajeunesse 2011](#_ENREF_8)). Our meta-analysis included both completely randomised designs and matched designs in the form of randomised block or systematic designs *sensu* [Hurlbert (1984](#_ENREF_6)). For a control group of old-growth forest and a treatment group of restored forest, the effect-size variance is estimated from group means ͞*x_i_* , standard deviations *s_i_*, and sample sizes *n_i_*. The variance estimate for a completely randomised study design is: $Var_{\ln R}=s_{1}^{2}/\left(n_{1}\bar{x}_{1}^{2}\right)+s_{2}^{2}/\left(n_{2}\bar{x}_{2}^{2}\right)$. The variance estimate for a matched study design requires a further subtraction from this estimate, of an amount equal to $2rs_{1}s_{2}/\left(\bar{x}_{1}\bar{x}_{2}n\right)$, where *r* is the coefficient of correlation between control and treatment measurements ([Lajeunesse 2011](#_ENREF_8)). Randomised blocks or systematic groups that contain old-growth and secondary forest stands generally have low replication in ecological studies, due to the time and expense demanded by biodiversity surveys. Studies for our meta-analysis frequently reported replications of two and three matches (i.e. randomised blocks or systematic pairs) of old-growth and restored forests. Such low sample sizes make for nonsense estimations of *r*, and consequently of variances, for matched designs. In practice, incorporating these nonsense estimates for matched designs has the effect of reducing variance by orders of magnitude. Excluding studies with matched designs from the analysis, however, would greatly reduce the sample size of the meta-analysis (56 out of the 90 studies had matched study designs).

In meta-regression with weightings on sample size or variance, the presence of large-sample outliers can substantially influence parameter estimates ([Fuller & Hester 1999](#_ENREF_5)). Discarding them from the analysis has the unsatisfactory consequence of a substantial decrease in power due to losing the studies most likely to have highest precision ([Fuller & Hester 1999](#_ENREF_5)).

In lieu of excluding studies for want of more information on observed or expected variance, we set stringent criteria for including studies in the meta-analysis, based on the quality of their designs with respect to distinguishing treatment effects from unmeasured variation. Meta-analyses in ecology frequently use unweighted effect sizes, particularly in the absence of satisfactory measures of observed or expected variance ([Benayas et al. 2009](#_ENREF_1); [Koricheva & Gurevitch 2014](#_ENREF_7); [Martin et al. 2013](#_ENREF_9); [Moreno-Mateos et al. 2012](#_ENREF_11); [Putz et al. 2012](#_ENREF_12)).[Cardinale et al. (2006](#_ENREF_3)) and [Marvier et al. (2007](#_ENREF_10)) found little difference between the results of weighted and unweighted meta-analyses. Unweighted meta-regression is often more robust, because it does not use potentially misleading estimation of error variances ([Fletcher & Dixon 2012](#_ENREF_4)).

Benayas, J. M. R., A. C. Newton, A. Diaz, and J. M. Bullock. 2009. Enhancement of Biodiversity and Ecosystem Services by Ecological Restoration: A Meta-Analysis. Science **325**:1121-1124.

Borenstein, M. B., L. V. Hedges, J. P. T. Higgins, and H. R. Rothstein 2009. Introduction to Meta-Analysis. John Wiley & Sons, Ltd, Chichester, UK.

Cardinale, B. J., D. S. Srivastava, J. E. Duffy, J. P. Wright, A. L. Downing, M. Sankaran, and C. Jouseau. 2006. Effects of biodiversity on the functioning of trophic groups and ecosystems. Nature **443**:989-992.

Fletcher, D., and P. M. Dixon. 2012. Modelling data from different sites, times or studies: weighted vs. unweighted regression. Methods in Ecology and Evolution **3**:168-176.

Fuller, J. B., and K. Hester. 1999. Comparing the sample-weighted and unweighted meta-analysis: An applied perspective. Journal of Management **25**:803-828.

Hurlbert, S. H. 1984. Pseudoreplication and the Design of Ecological Field Experiments. Ecological Monographs **54**:187-211.

Koricheva, J., and J. Gurevitch. 2014. Uses and misuses of meta-analysis in plant ecology. Journal of Ecology **102**:828-844.

Lajeunesse, M. J. 2011. On the meta-analysis of response ratios for studies with correlated and multi-group designs. Ecology **92**:2049-2055.

Martin, P. A., A. C. Newton, and J. M. Bullock. 2013. Carbon pools recover more quickly than plant biodiversity in tropical secondary forests. Proceedings of the Royal Society B-Biological Sciences **280**.

Marvier, M., C. McCreedy, J. Regetz, and P. Kareiva. 2007. A meta-analysis of effects of Bt cotton and maize on nontarget invertebrates. Science **316**:1475-1477.

Moreno-Mateos, D., M. E. Power, F. A. Comin, and R. Yockteng. 2012. Structural and Functional Loss in Restored Wetland Ecosystems. Plos Biology **10**.

Putz, F. E., P. A. Zuidema, T. Synnott, M. Pena-Claros, M. A. Pinard, D. Sheil, J. K. Vanclay, P. Sist, S. Gourlet-Fleury, B. Griscom, J. Palmer, and R. Zagt. 2012. Sustaining conservation values in selectively logged tropical forests: the attained and the attainable. Conservation Letters **5**:296-303.
